# Preeclampsia-exposed children’s heart rate variability 8–12 yr after index pregnancy: FINNCARE study

**DOI:** 10.1152/ajpheart.00540.2023

**Published:** 2023-11-03

**Authors:** Michelle Renlund, Mikko P. Tulppo, Anni Kivelä, Hannele Laivuori, Seppo Heinonen, Tiina Jääskeläinen, Taisto Sarkola

**Affiliations:** ^1^Children’s Hospital, Pediatric Research Center, University of Helsinki and Helsinki University Hospital, Helsinki, Finland; ^2^Minerva Foundation Institute for Medical Research, Helsinki, Finland; ^3^Research Unit of Biomedicine and Internal Medicine, Medical Research Center Oulu, Oulu University Hospital and University of Oulu, Oulu, Finland; ^4^Medical and Clinical Genetics, University of Helsinki and Helsinki University Hospital, Helsinki, Finland; ^5^Department of Obstetrics and Gynecology, Faculty of Medicine and Health Technology, Tampere Center for Child, Adolescent, and Maternal Health Research, Tampere University Hospital and Tampere University, Tampere, Finland; ^6^Department of Obstetrics and Gynecology, Helsinki University Hospital, Helsinki, Finland; ^7^Department of Food and Nutrition, University of Helsinki, Helsinki, Finland

**Keywords:** adiposity, blood pressure, cardiovascular disease, heart rate variability, preeclampsia

## Abstract

Preeclampsia is related with elevated systolic blood pressure (SBP) in children. We studied if preeclampsia-exposed (PE) children develop alterations in heart rate variability (HRV) and if this is reflected in their blood pressure (BP), as well as overall associations with body size and composition, gestational and perinatal factors. We examined 182 PE (46 early-onset PE) and 85 unexposed (non-PE) children 8–12 yr after preeclampsia exposure. HRV monitoring was performed 5 min in supine followed by 5 min in standing position and compared with office, 24-h ambulatory, and central BPs in relation to body anthropometrics and composition, gestational, and perinatal data. There were no major differences in HRV between PE and non-PE children. HRV in supine position was strongly associated with office and ambulatory heart rates (HRs), and HR was independently associated with office BPs. However, HRV was not related with office or 24-h SBP and PP, nor with elevated SBP in PE compared with non-PE children [adjusted mean differences for office and 24-h SBP 4.8 (*P* < 0.001) and 2.5 mmHg (*P* = 0.049), respectively]. In supine position, high-frequency (HF) power [β, −0.04 (95% CI −0.06 to −0.01)], root mean square of successive differences in R-R intervals (rMSSD) [−0.015 (−0.028 to −0.002)], and the ratio of low-frequency (LF) to HF power [0.03 (0.01–0.04)] were independently associated with child fat mass. LF and HF power and rMSSD displayed independent inverse associations with child age. There were no significant associations between child HRV and gestational and perinatal factors. During prepuberty, the HRV in children with PE is similar to that in non-PE children. Elevated SBP following preeclampsia exposure is not related with HRV. Child adiposity could be related to decreased cardiac vagal tone.

**NEW & NOTEWORTHY** Heart rate variability in preadolescent children exposed to preeclampsia in utero is no different from age-matched controls. Preeclampsia-exposed children’s elevated SBP is not related to alterations in heart rate variability, which is a noninvasive measure of the modulation of heart rate by autonomic tone. However, childhood adiposity might be coupled with diminished cardiac vagal tone.

## INTRODUCTION

Heart rate variability (HRV) is a noninvasive measure of the modulation of heart rate (HR) by autonomic tone (1). Altered cardiovascular autonomic regulation, more specifically lowered HRV, has been linked to cardiovascular risk factors and mortality long term (1, 2). In children, cardiovascular risk factors, including obesity and systolic blood pressure (SBP), are associated with altered HRV (3). Children and adolescents with hypertension or high blood pressure (BP), but still within the normal range, show a trend toward sympathetic predominance (4), as well as reduced autonomic regulation and impaired baroreflex sensitivity (5). Niemirska et al. (6) examined adolescents with primary hypertension after successful antihypertensive treatment, but despite treatment, altered cardiovascular rhythmicity persisted as a potential primary indicator of aberrancy.

Preterm birth and born small for gestational age (SGA) are conditions coupled with increased sympathoadrenal activity in children (7). Prematurity has also been associated with modified autonomic nervous system function through alterations in HRV (8), and prematurity is linked with cardiometabolic risk factors in adolescence (9). Preterm birth is common in early-onset preeclampsia and other hypertensive disorders of pregnancy (HDPs)(10). Preeclampsia is characterized by new-onset hypertension and proteinuria after 20 gestational wk or new-onset preeclampsia-associated signs in the absence of proteinuria, and it affects up to 10% of all pregnancies (11). Plasma epinephrine levels have been related with elevated 24-h SBP in preeclampsia-exposed (PE) children, but similar associations were also observed for unexposed (non-PE) children in the same study (12). We have recently reported elevated office, 24-h ambulatory, and central BPs in PE children (13).

To the best of our knowledge, there are no previous studies investigating children’s HRV following preeclampsia exposure in utero. Our hypothesis was that PE children develop alterations in HRV reflecting cardiac autonomic dysfunction, and that this is reflected in their elevated BP. We aimed to prospectively compare PE children 8–12 yr after index pregnancy with age-matched children without preeclampsia or other HDP. Furthermore, we aimed to evaluate relations between HRV and BP, body anthropometrics and composition at follow-up, and maternal, gestational, and perinatal factors.

## MATERIALS AND METHODS

### Study Design, Sample, and Setting

The Finnish Genetics of Preeclampsia Consortium (FINNPEC) multicenter study cohort recruited prospectively between 2008 and 2011 nulli- or multiparous PE and non-PE white Caucasian women, as well as their partners and newborns (14). The FINNPEC cohort defined preeclampsia as hypertension and proteinuria arising after 20 wk of gestation: SBP ≥ 140 mmHg and/or diastolic BP (DBP) ≥ 90 mmHg, and urinary excretion of ≥0.3 g of protein in a 24-h specimen, or 0.3 g/L, or two ≥1 + readings on dipstick.

In the FINNCARE study, we recalled 366 out of 471 FINNPEC PE families and 233 out of 307 FINNPEC non-PE families living in the Hospital district of Helsinki and Uusimaa (NCT04676295) (15). Study families were examined prospectively in a cohort study setting from June 2019 to June 2022 at the Clinical Trial Unit at the Children’s Hospital (Helsinki, Finland). One hundred and eighty-two PE and 85 non-PE children’s CVD risk profiles were prospectively assessed. We excluded study families where the mother had ongoing pregnancy or lactation, multiple pregnancy, or was not able to communicate in Finnish. Exclusion criteria for non-PE families also included the following: PE or other HDP, chronic hypertension, gestational diabetes, and/or diabetes during or after the index pregnancy. Background characteristics were compared between participating and nonparticipating PE mothers from the Hospital district of Helsinki and Uusimaa FINNPEC cohort to assess possible recruitment bias. No significant differences were found in major maternal, gestational, and child perinatal factors (results not shown). Signed informed consent was gathered from all study participants, and the FINNCARE study protocol was approved in December 2018 by the Ethics Committee of the Hospital District of Helsinki and Uusimaa (HUS/3347/2018).

### Index Pregnancy and Questionnaire Data

Data on index pregnancy were gathered from the FINNPEC database (14). Early-onset preeclampsia was defined as diagnosis or delivery before 34^0/7^ gestational wk. Children were classified as premature when delivered before 37^0/7^ gestational wk. *Z*-scores were calculated for birth height, weight, and head circumference (16). SGA was defined as birth weight <−2 standard deviations (SDs). We collected information on family health history and current household income through standard questionnaires at the FINNCARE study visits.

### Heart Rate Variability

An HR monitor (Bittium Faros 360, Oulu, Finland) was placed on the study participant’s chest following a 30-min rest in the supine position to assess HRV. Monitoring was then performed during spontaneous and quiet breathing at rest in the supine position for 5 min and directly continued for an additional 5 min in a still-standing position. A 4-min supine position interval starting 30 s from beginning, and a 4-min standing position interval at the end of the total 10-min recording were then analyzed with the operator (M.P.T) blinded to study subject characteristics (Hearts 1.2, University of Oulu, Oulu, Finland). Artifacts and ectopic beats were removed and replaced by the local average. However, sequences with ≥10 consecutive beats of noise or ectopic beats were deleted. The R-R interval series with ≥80% accepted data were included in the analyses. Two-hundred and thirty-seven PE and non-PE children provided R-R interval recordings, with 230 (97%) having eligible HRV data for both phases, supine and standing intervals. Mean HR, average R-R interval, standard deviation of all R-R intervals over the total 4-min period (RR-SD, ms), root mean square of successive differences in R-R intervals (rMSSD, ms), spectral power densities (fast-Fourier transformations) at low-frequency (LF, 0.04–0.15 Hz, ms^2^) and high-frequency (HF, 0.15–0.40 Hz, ms^2^) components of HRV, and their ratio (LF:HF) were analyzed (17). Coefficient of component variance (CCV%) for LF and HF power, respectively, was calculated as square root of power divided by mean R-R interval. Furthermore, we assessed autonomic nervous system function also as parameter change from supine to standing position since LF:HF ratio may estimate sympathetic modulation throughout the sympathetic activation mediated by orthostatic stimulus (18). HF power and rMSSD are regarded as markers of parasympathetic regulation and vagal tone, whereas LF power is a marker of both parasympathetic and sympathetic modulation (17). LF power has also been suggested to even more reflect modulation of cardiac autonomic outflow by baroreflexes than by sympathetic tone (19). LF:HF ratio has been considered a marker of sympathovagal balance (20), but this perception has been challenged and the interpretation is still under debate (21).

### Blood Pressure and Pulse Wave Velocity

BP was measured from the nondominant arm at the office with Omron HBP-1300 or HBP-1320 devices and monitored during 24-h with an oscillometric Schiller BR-102 plus device. Ambulatory BP (ABP) was assessed at 30-min intervals during daytime and at 1-h intervals during nighttime. One investigator analyzed the ambulatory BP data in agreement with BP diaries and ultimately rejected 16 daytime and 18 nighttime ABP registrations due to less than 65% valid measurements (22). Mean arterial pressure (MAP) was calculated from DBP + 1/3(SBP − DBP) and pulse pressure (PP) as the difference between SBP and DBP.

We measured carotid-femoral (CF-PWV) and carotid-radial pulse wave velocity (CR-PWV) at rest with the study participant in a supine position (Complior Analyze, Alam Medical, Saint-Quentin-Fallavier, France). In analyses, the mean of two measurements was used, and we multiplied the CF distance by 0.8. We obtained central BPs from the Complior device and its direct analysis of the carotid waveform and calibration with diastolic and mean office brachial BP.

### Anthropometrics

We used a Seca 285 scale and stadiometer (Seca GmBH & Co, Hamburg, Germany) to measure height and weight to the nearest 0.1 cm and 0.05 kg, respectively. Waist, hip, and thoracic circumferences were estimated to the closest 0.1 cm with a measuring tape. *Z*-scores based on age and sex were generated for height, weight, and body mass index (BMI), and for weight separately for height and sex based on national data (23). Lean body mass, skeletal muscle mass, fat mass, and body fat percentage were computed with bioelectrical impedance (InBody 720, InBody Bldg, Korea). Haycock’s formula was used to calculate body surface area (BSA).

### Data Analysis

We present results as means (SD), medians (interquartile ranges), and counts (percentages) as appropriate. Histograms, normality tests, and skewness were used to assess normality. Group differences were calculated using independent samples *t* test or Mann–Whitney *U* test for numerical data, and Pearson χ^2^ or two-tailed Fisher’s exact test for categorical data. LF and HF power, LF:HF ratio, and rMSSD were transformed into natural logarithm (ln) in analyses because of skewed distributions. Delta (change) was calculated for HRV variables by mathematically subtracting supine values from standing values for each study subject.

Potential predictors for children’s HRV variables were analyzed with univariate linear regression analyses and we report in results only associations with a *P* < 0.015 when considering the number of tests in univariate regression analyses and possibility of type 1 error. Multiple linear regression models were then built to assess potential predictors’ combined influence on children’s HRV in supine position. The models were checked for linearity, normality, homoscedasticity, and independence. Multicollinearity was evaluated with a variance influence factor <2.5 and collinearity tolerance >0.3 was deemed appropriate. Twenty-four-hour SBP and PP ANCOVA models have previously been analyzed and published by us (13), and here we checked the influence of HRV variables on the adjusted office and 24-h SBP and PP mean differences between PE and non-PE children. *P* < 0.05 was considered significant and two-tailed tests were used in analyses. Statistical analysis was performed using SPSS v. 27 (IBM).

## RESULTS

### Perinatal and Child Background Characteristics

One-hundred and eighty-two PE children (46 early-onset based on diagnosis, 25 on delivery) and 85 non-PE children were examined at the follow-up visit (Table 1). Preterm birth and SGA were common among PE children, as expected, but there were no major differences in body anthropometrics and composition. Office BPs were higher among PE versus non-PE children.

**Table 1.** Child characteristics

	Non-PE	PE	Early-Onset PE (Diagnosis< 34^0/7^ wk)	Late-Onset PE (Diagnosis≥ 34^0/7^ wk)	*P* Value (PE vs. Non-PE)	Mean Difference (95% CI)	*P* Value [Early (dg) vs. Non-PE]	Mean Difference (95% CI)	*P* Value [Late (dg) vs. Non-PE]	Mean Difference (95% CI)
*n*	85	182	46	136						
Perinatal and follow-up characteristics										
Premature, *n* (%)	4 (4.7)	60 (33.0)	42 (91.3)	18 (13.2)	**<0.001**		**<0.001**		**0.039**	
SGA, *n* (%)	0 (0)	32 (17.6)	15 (32.6)	17 (12.5)	**<0.001**		**<0.001**		**<0.001**	
Age, yr	11.2 (1.0)	11.6 (1.1)	11.6 (1.2)	11.6 (1.1)	**0.004**	0.4 (0.1–0.7)	**0.035**	0.4 (0–0.8)	**0.006**	0.4 (0.1–0.7)
Girls, *n* (%)	42 (49.4)	99 (54.4)	25 (54.3)	74 (54.4)	0.447		0.590		0.469	
Body height, cm	149.3 (8.4)	151.2 (10.1)	148.7 (9.1)	152.0 (10.3)	0.132	1.9 (−0.6 to 4.4)	0.713	−0.6 (−3.7 to 2.5)	**0.040**	2.7 (0.1–5.4)
Height *z*-score	0.15 (0.89)	0.05 (1.10)	−0.30 (1.18)	0.17 (1.06)	0.450	−0.10 (−0.37 to 0.17)	**0.015**	−0.45 (−0.81 to −0.09)	0.918	0.01 (−0.26 to 0.29)
Body weight, kg	39.4 [10.4]	40.3 [17.1]	41.2 [18.5]	40.1 [16.0]	0.427	1.0 [−1.6 to 3.5]	0.477	1.2 [−2.5 to 5.5]	0.490	0.9 [−1.7 to 3.5]
Weight *z*-score (height)	−0.13 (0.95)	−0.22 (1.10)	0.16 (0.97)	−0.35 (1.11)	0.502	−0.09 (−0.36 to 0.18)	0.102	0.29 (−0.06 to 0.63)	0.129	−0.22 (−0.51 to 0.06)
Body surface area, m^2^†	1.30 (0.23)	1.33 (0.22)	1.33 (0.24)	1.33 (0.22)	0.228	0.03 (−0.02 to 0.08)	0.438	0.03 (−0.05 to 0.11)	0.255	0.03 (−0.02 to 0.08)
Lean mass bioimpedance, kg	32.6 (5.7)	33.7 (7.2)	32.9 (6.6)	33.9 (7.4)	0.175	1.1 (−0.5 to 2.7)	0.737	0.4 (−1.8 to 2.6)	0.125	1.4 (−0.4 to 3.1)
Adiposity measures										
Waist circumference, cm	63.0 [8.9]	64.1 [11.0]	67.0 [12.2]	63.2 [10.5]	0.331	1.0 [−1.0 to 3.0]	0.109	2.7 [−0.5 to 5.9]	0.605	0.5 [−1.5 to 2.5]
Waist-hip ratio	0.82 (0.05)	0.83 (0.06)	0.85 (0.07)	0.82 (0.06)	0.582	0 (−0.01 to 0.02)	**0.038**	0.02 (0–0.05)	0.737	0 (−0.02 to 0.01)
BMI, kg/m^2^	17.8 [3.1]	17.6 [4.1]	18.6 [4.7]	17.5 [4.0]	0.993	−0.01 [−0.71 to 0.70]	0.215	0.64 [−0.39 to 1.82]	0.571	−0.19 [−0.93 to 0.50]
BMI *z*-score	−0.02 (0.97)	−0.13 (1.08)	0.14 (1.05)	−0.22 (1.08)	0.443	−0.11 (−0.38 to 0.17)	0.381	0.16 (−0.20 to 0.52)	0.175	−0.20 (−0.48 to 0.09)
Fat mass, kg	6.3 [5.7]	6.6 [7.1]	7.5 [9.1]	6.6 [5.6]	0.914	0.1 [−1.0 to 1.1]	0.291	0.9 [−0.7 to 2.9]	0.734	−0.2 [−1.3 to 0.9]
Body fat percentage, %	19.0 (8.3)	18.9 (8.9)	21.2 (9.6)	18.1 (8.5)	0.920	−0.1 (−2.4 to 2.1)	0.170	2.2 (−1.0 to 5.4)	0.435	−0.9 (−3.2 to 1.4)
Office blood pressure									
SBP, mmHg	109.6 (7.4)	114.9 (9.7)	115.2 (9.4)	114.8 (9.8)	**<0.001**	5.3 (3.2–7.4)	**<0.001**	5.6 (2.7–8.6)	**<0.001**	5.2 (2.9–7.5)
DBP, mmHg	68.6 (5.8)	70.4 (6.2)	71.2 (6.5)	70.1 (6.1)	**0.023**	1.8 (0.3–3.4)	**0.018**	2.7 (0.5–4.9)	0.064	1.5 (−0.1 to 3.2)
PP, mmHg	41.0 (6.6)	44.2 (7.7)	43.8 (8.7)	44.4 (7.4)	**0.001**	3.2 (1.3–5.1)	**0.045**	2.7 (0.1–5.4)	**<0.001**	3.4 (1.4–5.3)
HR, beats/min	76.1 (11.3)	76.4 (10.2)	74.8 (9.7)	76.9 (10.3)	0.830	0.3 (−2.5 to 3.1)	0.533	−1.3 (−5.2 to 2.7)	0.585	0.8 (−2.1 to 3.8)
SBP *z*-score	0.63 (0.74)	1.01 (0.81)	1.13 (0.76)	0.97 (0.82)	**<0.001**	0.38 (0.18–0.59)	**<0.001**	0.50 (0.23–0.78)	**0.002**	0.34 (0.13–0.56)
DBP *z*-score	0.63 (0.58)	0.80 (0.60)	0.92 (0.64)	0.76 (0.58)	**0.027**	0.17 (0.02–0.33)	**0.009**	0.29 (0.07–0.51)	0.100	0.13 (−0.03 to 0.29)

Values are means (SD) and medians [interquartile ranges], median differences (95% confidence intervals, CI); *n*, number of subjects. †Calculated with Haycock’s formula. PE, preeclampsia exposed; non-PE, unexposed; BMI, body mass index; SBP, systolic blood pressure; DBP, diastolic blood pressure; PP, pulse pressure; HR, heart rate; dg, diagnosis; SGA, small for gestational age (birth weight < −2SD); premature, birth <37^0/7^ gestational wk. Independent samples *t* test for normally distributed numerical data. Mann–Whitney *U* test for nonnormal distribution and Pearson χ^2^ test or Fisher’s exact test for categorical data. Significant *P* < 0.05, shown in boldface.

### Heart Rate Variability in PE and non-PE Children at 8–12 yr Follow-Up

**Figure 1.**
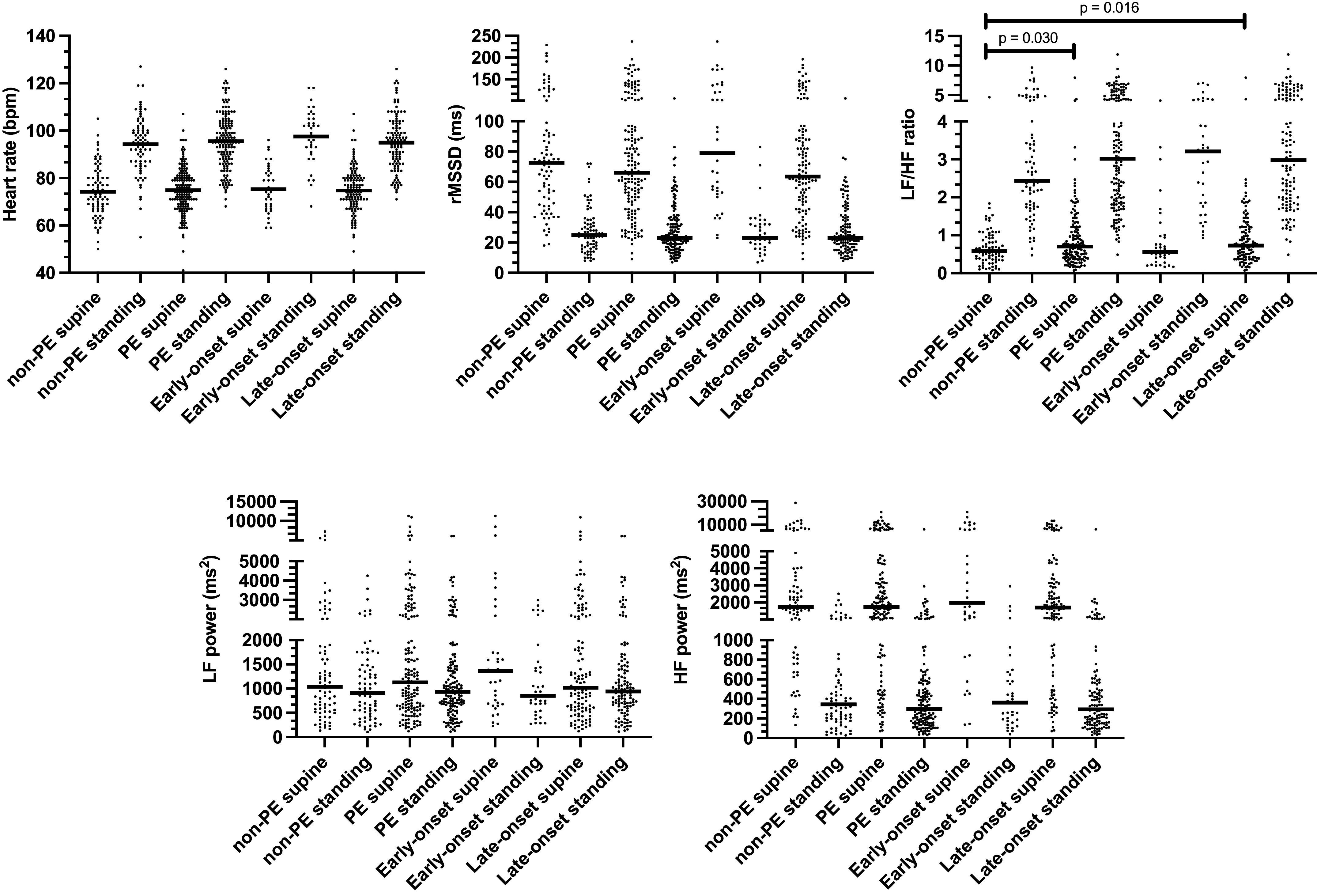
Children’s heart rate variability at follow-up in supine and standing positions. early onset PE, preeclampsia diagnosis before 34^0/7^ gestational wk; HF power, high-frequency power of R-R intervals from spectral band 0.15–0.4 Hz (fast-Fourier transformation); LF power, low-frequency power of R-R intervals from spectral band 0.04–0.15 Hz (fast-Fourier transformation); late-onset PE, preeclampsia diagnosis at or after 34^0/7^ gestational wk; non-PE, unexposed control group; PE, preeclampsia-exposed group; rMSSD, root mean square of successive differences in R-R intervals. Horizontal line shows median value for all other variables except heart rate showing mean value. *P* < 0.05, significance threshold.

LF:HF ratio in supine position was borderline elevated among PE versus non-PE children {mean difference 0.23 [95% confidence interval (CI) 0.02–0.45]; *P* = 0.030; Fig. 1, Supplemental Table S1; all Supplemental material is available at https://doi.org/10.6084/m9.figshare.24304450.v1}, and this was attributed to the late-onset PE group [mean difference 0.27 (95% CI 0.05–0.49); *P* = 0.016]. No other differences in HRV variables between PE (including early- and late-onset PE) and non-PE children were found (results not shown for early and late PE by delivery definition). CCV%s for LF and HF power were no different between groups (including early- and late-onset PE; results not shown).

### Predictors of Heart Rate Variability in PE and Non-PE Children

HR in supine position was in univariate analyses inversely associated with height *z*-score, while positively associated with weight *z*-score, adiposity measures, office BPs, as well as with office and 24-h (including daytime and nighttime) HRs (Supplemental Table S2). rMSSD, LF, and HF power in supine position showed clear inverse associations with office and day- and nighttime HR. Furthermore, rMSSD and HF power in supine position showed significant inverse relations with child age at follow-up, office BPs, several anthropometric (weight, BSA, LBM, muscle mass, and thoracic circumference) and adiposity measures [waist circumference, BMI (and its *z*-score), fat mass and body fat percentage], as well as rMSSD with office central DBP. LF power in supine position was related with male sex and inversely related with age, while LF:HF ratio displayed positive associations with several anthropometric (weight, BSA, LBM, muscle mass, and thoracic circumference) and adiposity measures (BMI, fat mass, waist circumference, and waist-hip ratio). No associations were seen between HRV variables and maternal (age at delivery, prepregnancy BMI, parity, smoking during pregnancy, and gestational and follow-up office BPs) and perinatal parameters (gestational time at delivery, prematurity, SGA, birth height, weight, and head circumference). Children’s HRV variables in standing position displayed no associations, and the change in HRV variables from supine to standing position showed no associations in univariate regressions.

For HR in multiple linear regression analyses, child height *z*-score and office SBP at follow-up were significant independent predictors (model’s adjusted *R*^2^ = 0.096, *P* < 0.001; Table 2). Both rMSSD and HF power were in models independently inversely associated with child age, fat mass, and 24-h HR (rMSSD model’s adjusted *R*^2^ = 0.184, *P* < 0.001; and HF power model’s adjusted *R*^2^ = 0.183, *P* < 0.001). LF power was independently inversely related with child age and 24-h HR (model’s adjusted *R*^2^ = 0.134, *P* < 0.001). LF:HF ratio was positively associated with child fat mass (model’s adjusted *R*^2^ = 0.065, *P* < 0.001). Preeclampsia exposure displayed only borderline significance in the LF:HF ratio model (standardized β coefficient, 0.133, *P* = 0.039).

**Table 2.** Multiple linear regression models for all children’s heart rate variability in supine position

Heart Rate Variability/Predictor	Unstandardized β (95% CI)	Standardized β	*P* Value	Adjusted *R*^2^	Model *P* Value
HR					
Constant	38.94 (22.74–55.15)			0.096	**<0.001**
Preeclampsia exposure (0 = no, 1 = yes)	−1.12 (−3.84 to 1.61)	−0.052	0.421		
Child height *z*-score at follow-up	−2.20 (−3.39 to −1.01)	−0.232	**<0.001**		
Child office SBP at follow-up, mmHg	0.33 (0.18–0.47)	0.286	**<0.001**		
ln rMSSD					
Constant	7.98 (6.66–9.31)			0.184	**<0.001**
Preeclampsia exposure (0 = no, 1 = yes)	−0.06 (−0.23 to 0.10)	−0.052	0.452		
Child age at follow-up, yr	−0.12 (−0.21 to −0.04)	−0.210	**0.004**		
Child fat mass at follow-up, kg	−0.015 (−0.028 to −0.002)	−0.162	**0.022**		
Child 24-h HR, beats/min	−0.03 (−0.04 to −0.02)	−0.342	**<0.001**		
ln LF power					
Constant	12.62 (10.60–14.64)			0.134	**<0.001**
Preeclampsia exposure (0 = no, 1 = yes)	0.10 (−0.16 to 0.36)	0.056	0.432		
Child age at follow-up, yr	−0.25 (−0.37 to −0.12)	−0.279	**<0.001**		
Child 24-h HR, beats/min	−0.04 (−0.05 to −0.02)	−0.300	**<0.001**		
ln HF power					
Constant	14.52 (11.93–17.10)			0.183	**<0.001**
Preeclampsia exposure (0 = no, 1 = yes)	−0.13 (−0.46 to 0.20)	−0.053	0.445		
Child age at follow-up, yr	−0.24 (−0.40 to −0.07)	−0.206	**0.005**		
Child fat mass at follow-up, kg	−0.04 (−0.06 to −0.01)	−0.195	**0.006**		
Child 24-h HR, beats/min	−0.05 (−0.07 to −0.03)	−0.318	**<0.001**		
ln LF:HF ratio					
Constant	−1.83 (−1.05 to −0.62)			0.065	**<0.001**
Preeclampsia exposure (0 = no, 1 = yes)	0.22 (0.01–0.43)	0.133	**0.039**		
Child fat mass at follow-up, kg	0.03 (0.01–0.04)	0.204	**0.002**		

Values are β (95% confidence intervals, CI). HR, heart rate; SBP, systolic blood pressure; rMSSD, root mean square of successive differences in R-R intervals; LF power, low-frequency power of R-R intervals from spectral band 0.04–0.15 Hz (fast-Fourier transformation); HF power, high-frequency power of R-R intervals from spectral band 0.15–0.4 Hz (fast-Fourier transformation). Significant *P* values < 0.05, shown in boldface.

### Adjusted Differences for HRV and BP Variables between PE and Non-PE Children

Adjusted office and 24-h SBP and PP mean differences between PE and non-PE children remained significant or borderline significant when adding rMSSD (supine) or LF:HF ratio (supine and standing, respectively) together with child body fat percentage at follow-up (Table 3).

**Table 3.** Adjusted mean differences for office and 24-h SBP and PP in preeclampsia-exposed versus -unexposed children

Outcome/Covariates	Mean Difference (95% CI)	*P* Value
Office SBP		
Unadjusted mean difference	5.3 (3.2–7.4)	**<0.001**
*Model 1*: %Child fat at follow-up	5.4 (3.1–7.7)	**<0.001**
*Model 2*: %Child fat +ln rMSSD (supine)	4.7 (2.4–7.0)	**<0.001**
*Model 3*: %Child fat +ln LFHF ratio (supine)	4.6 (2.2–6.9)	**<0.001**
*Model 4*: %Child fat +ln LFHF ratio (standing)	4.8 (2.4–7.2)	**<0.001**
24-h SBP		
Unadjusted mean difference	2.9 (0.4–5.3)	**0.024**
*Model 1*: %Child fat at follow-up	2.9 (0.4–5.3)	**0.023**
*Model 2*: %Child fat+ln rMSSD (supine)	2.4 (−0.09 to 4.9)	0.059
*Model 3*: %Child fat+ln LFHF ratio (supine)	2.4 (−0.12 to 4.9)	0.062
*Model 4*: %Child fat+ln LFHF ratio (standing)	2.1 (−0.4 to 4.6)	0.104
Office PP		
Unadjusted mean difference	3.2 (1.3–5.1)	**0.001**
*Model 1*: %Child fatat follow-up	3.5 (1.4–5.5)	**<0.001**
*Model 2*: %Child fat+ln rMSSD (supine)	3.5 (1.4–5.5)	**0.001**
*Model 3*: %Child fat+ln LFHF ratio (supine)	3.3 (1.3–5.4)	**0.002**
*Model 4*: %Child fat+ln LFHF ratio (standing)	3.6 (1.5–5.7)	**<0.001**
24-h PP		
Unadjusted mean difference	3.7 (1.9–5.4)	**<0.001**
*Model 1*: %Child fat at follow-up	3.6 (1.5–5.6)	**<0.001**
*Model 2*: %Child fat+ln rMSSD (supine)	3.4 (1.3–5.4)	**0.001**
*Model 3*: %Child fat+ln LFHF ratio (supine)	3.3 (1.2–5.4)	**0.002**
*Model 4*: %Child fat+ln LFHF ratio (standing)	3.2 (1.2–5.3)	**0.002**

Values are mean differences (95% confidence intervals, CI). SBP, systolic blood pressure; PP, pulse pressure; rMSSD, root mean square of successive differences in R-R intervals; LF power, low-frequency power of R-R intervals from spectral band 0.04–0.15 Hz (fast-Fourier transformation); HF power, high LF and HF. Significant *P* values < 0.05, shown in boldface.

## DISCUSSION

This study found no significant alterations in HRV in prepubertal children with PE compared with those without. HRV variables were strongly associated with office and ambulatory HRs indicating valid and concordant methodology. Associations between HR and office BPs are likely explained by simultaneous situational factors during HRV monitoring. However, no relations between HRV and elevated office and 24-h SBP and PP in PE children were found. HF power and rMSSD in supine position were negatively associated, and LF:HF ratio was positively associated, with adiposity measures. No associations between HRV during standing position or between HRV change from supine to standing and child parameters at follow-up, maternal, gestational, and perinatal factors variables were found. Taken together, HRV alterations are not associated with PE children’s elevated BP profile during preadolescent age.

Borderline significantly higher LF:HF ratio among the late-onset PE children was the only noted difference in HRV between PE and non-PE children. This could indicate impaired sympathovagal balance and increased sympathetic activity, although, the significance of the variable is debated (21). The relevance of this finding is questionable and does not seem to be related with blood pressure control as early-onset PE children did not display any alterations in the LF:HF ratio, and early-onset preeclampsia is associated with a more severe form of preeclampsia than late onset. A trend toward higher LF:HF ratio in adolescent males with higher BP (4) and significantly higher LF:HF ratio among 11–14-yr-old children with elevated BP has previously been reported (5). In preadolescent children, presence of cardiovascular risk factors has also been associated with higher LF:HF ratio (3). To our knowledge, PE children’s HRV has not previously been assessed and this study therefore brings new information to the literature. Our reported HRV variable levels in our relatively low cardiovascular-risk child study population are similar or somewhat higher than previously reported in a population of 465 healthy Finnish children aged 6–8 yr assessed during similar conditions (24).

We have recently reported elevated BPs in this same population of 8–12-yr-old preadolescent PE children compared with non-PE children (13). In the present study, HF power and rMSSD showed negative associations with office BP variables suggesting a role of vagal tone in BP regulation. In contrast, none of the HRV variables were, however, associated with ambulatory BPs, and thus associated with diurnal BP levels. Moreover, HRV was not related with the observed difference in office or 24-h SBP (or PP) between PE and non-PE children suggesting a limited role of the autonomic nervous system in PE children’s elevated BP. Our results are thus in contrast with previous studies reporting altered autonomic activation in premature young adults (8) and premature and SGA-born children (7). Although preeclampsia is strongly associated with prematurity (10), and in the present study, one-fourth of PE children were born premature, PE children’s HRV was no different from non-PE children. In agreement with this, we found no statistically significant associations between HRV and any maternal, gestational, or perinatal factors. These differences between current and previous findings might be due to alterations in cardiac autonomic regulatory control becoming more evident later in adult age (8). In addition, urinary catecholamines, which are not direct indicators of sympathetic nervous tone, have been shown to be borderline elevated in prematurely born children (7). Our results then suggest that HRV, as a measure of the modulation of HR by autonomic tone, is not a key factor in the preadolescent PE children’s pathway to elevated BP that is likely influenced by maternal BP through shared genetics, in utero and/or postnatal lifestyle factors (25).

In the present study, children’s HF power and rMSSD demonstrated independent and negative associations with adiposity measures. This finding is consistent with previous reports in children with different cardiovascular risk factors (3) and obesity in particular (26–28). Furthermore, the finding that children’s LF:HF ratio was positively associated with adiposity measures is in agreement with previous reports in children with obesity although not previously reported in overweight children (26, 27). As our preadolescent PE population was primarily nonobese, we can only speculate if preeclampsia combined with more obesity would have been reflected in a more adverse HRV profile or if HRV alterations could emerge later in adolescence or early adulthood.

We found no clear independent associations between HRV variables and sex, and this is consistent with previous reports in healthy children (24, 29). Studies report parasympathetic HRV variables, such as rMSSD, not to be influenced by sex, but different standard deviations of R-R intervals to be higher in males (28, 30). In our population of 8–12-yr-old children, LF and HF power and rMSSD were negatively and independently predicted by child age as also previously reported (24, 30). Finley et al, (31) showed an increase in HRV during first 6 years of life followed by a decrease until 24 yr.

The prospective study design, age-matched control group, relatively large sample size, and comprehensive evaluation of HRV, BP, and maternal, gestational, and perinatal factors are considered strengths of this study. Limitations include the inability to assess associations with race and absence of postnatal child growth data.

In conclusion, PE children at mean 11 yr of age display no clear alterations in HRV compared with age-matched controls. PE children’s elevated SBP is not related with alterations in HRV, which reflects the modulation of HR by autonomic tone. Adiposity may, however, be related with decreased cardiac vagal tone during preadolescence.

## DATA AVAILABILITY

Data supporting the results are available upon reasonable request.

## SUPPLEMENTAL DATA

10.6084/m9.figshare.24304450.v1 Supplemental Tables S1 and S2: https://doi.org/10.6084/m9.figshare.24304450.v1.

## GRANTS

The FINNCARE AND FINNPEC studies received grants from Medicinska stiftelsen i Vasa (to M.R.), Finnish Foundation for Pediatric Research (to T.S.), The Medical Society of Finland (to T.S. and H.L.), Medicinska understödsföreningen Liv och Hälsa (to T.S.), Sigrid Juselius Foundation (to T.S.), Competitive state research funding of the expert responsibility areas of the Helsinki and Uusimaa Hospital (to T.S., S.H., and H.L.), Tampere University Hospital (to H.L.), Dorothea Olivia, Karl Walter and Jarl Walter Perklén Foundation (to T.S.), Juho Vainio Foundation (to T.J.), Päivikki and Sakari Sohlberg Foundation (to A.K. and H.L.), Academy of Finland (to H.L.), and Jane and Aatos Erkko Foundation (to H.L.).

## DISCLOSURES

No conflicts of interest, financial or otherwise, are declared by the authors.

## AUTHOR CONTRIBUTIONS

M.P.T., A.K., H.L., S.H., T.J., and T.S. conceived and designed research; M.R. and T.S. performed experiments; M.R. and M.P.T. analyzed data; M.R., M.P.T., and T.S. interpreted results of experiments; M.R. and T.S. drafted manuscript; M.R., M.P.T., A.K., H.L., S.H., T.J., and T.S. edited and revised manuscript; M.R., M.P.T., A.K., H.L., S.H., T.J., and T.S. approved final version of manuscript.
